# Cobdock: an accurate and practical machine learning-based consensus blind docking method

**DOI:** 10.1186/s13321-023-00793-x

**Published:** 2024-01-11

**Authors:** Sadettin Y. Ugurlu, David McDonald, Huangshu Lei, Alan M. Jones, Shu Li, Henry Y. Tong, Mark S. Butler, Shan He

**Affiliations:** 1 School of Computer Science, University of Birmingham, Edgbaston, Birmingham, B15 2TT UK; 2 AIA Insights Ltd, Birmingham, UK; 3 YaoPharma Co. Ltd., 100 Xingguang Avenue, Renhe Town, Yubei District, Chongqing, 401121 People’s Republic of China; 4 School of Pharmacy, University of Birmingham, Edgbaston, Birmingham, B15 2TT UK; 5 Centre for Artificial Intelligence Driven Drug Discovery, Macao Polytechnic University, R. de Luís Gonzaga Gomes, Macao, 5HV2+CP8 China

**Keywords:** Docking, Blind molecular docking, Global docking, Cross-docking, Reverse-docking, Inverse-docking, Consensus docking, Hybrid docking, Small molecule docking, Protein docking

## Abstract

Probing the surface of proteins to predict the binding site and binding affinity for a given small molecule is a critical but challenging task in drug discovery. Blind docking addresses this issue by performing docking on binding regions randomly sampled from the entire protein surface. However, compared with local docking, blind docking is less accurate and reliable because the docking space is too largetly sampled. Cavity detection-guided blind docking methods improved the accuracy by using cavity detection (also known as binding site detection) tools to guide the docking procedure. However, it is worth noting that the performance of these methods heavily relies on the quality of the cavity detection tool. This constraint, namely the dependence on a single cavity detection tool, significantly impacts the overall performance of cavity detection-guided methods. To overcome this limitation, we proposed **Co**nsensus **B**lind **Dock** (CoBDock), a novel blind, parallel docking method that uses machine learning algorithms to integrate docking and cavity detection results to improve not only binding site identification but also pose prediction accuracy. Our experiments on several datasets, including PDBBind 2020, ADS, MTi, DUD-E, and CASF-2016, showed that CoBDock has better binding site and binding mode performance than other state-of-the-art cavity detector tools and blind docking methods.

**Supplementary Information:**

The online version contains supplementary material available at 10.1186/s13321-023-00793-x.

## Introduction

Identifying the three-dimensional structures of protein-ligand complexes is an essential step in structure-based drug discovery. Technical developments in single co-crystal X-ray crystallography, cryo EM (electron microscopy) and nuclear magnetic resonance (NMR) spectroscopy are the reason for a much greater number of available high-resolution structures of proteins and protein-ligand complexes [1]; however, computational methods provide much faster and cheaper options to keep up with the rate at which new hit compounds, lead compounds, and therapeutic targets are found in drug discovery. Therefore, computational techniques like molecular docking are used as virtual screening tools in the structure-based drug discovery pipeline employed by the pharmaceutical and biotechnology industries [2].

Molecular docking is a computational method that predicts the binding pose and affinity of a ligand and a target protein structure [3]. It has become an indispensable tool in early-stage drug discovery and design, e.g., to discover drug hits in silico and to optimise hits as lead compounds. However, molecular docking methods require binding site information, i.e., a region on the target protein that binds to the ligand with specificity [4]. When the binding site information is unavailable, molecular docking methods must explore the whole protein surface to find a feasible binding pose. This strategy is also known as “blind docking” [5].

Blind docking methods have two subgroups: (i) Conventional blind docking, which uses molecular docking methods (such as, for example, AutoDock [6] and AutoDock Vina [7]) to search the entire surface of a target [5, 8–10] and (ii) Cavity detection-guided blind docking [11–14], which employs cavity detection tools such as P2Rank [15] and Fpocket [16] to determine potential binding pockets. Also, COACH-D [13] and GalaxySite [17] perform blind docking to identify binding sites instead of a cavity detection tool. After identifying the binding site, cavity detection-guided blind docking tools such as CB-Dock [12] and EDock [18] execute local docking at those predicted binding sites.

Conventional blind docking methods can perform poorly due to the large pose search areas [19]. Cavity detection-guided methods have improved the accuracy of blind docking by detecting the binding sites and then performing docking for a possible binding site. While cavity-guided blind docking methods can improve accuracy compared with conventional blind docking, often only a singular cavity detection tool is used and so their performance is highly dependent on the quality of that tool [20]. Due to the fact that a singular cavity detection cannot provide robust performance, it can result in a performance loss across a variety of benchmarks. To address these issues, Metapocket 2.0 combines more than one cavity detection tool [21]. However, there is still room to improve performance in blind docking through a consensus of multiple blind molecular docking and cavity detection tools.

To improve the performance of the blind docking method, we designed a **Co**nsensus **B**lind **Dock** (CoBDock) method. Unlike cavity detection-guided methods, which directly identify potential binding sites, CoBDock simultaneously extracts and integrates information from various docking methods and cavity detection tools in parallel. The intuition is that the parallel approach combines molecular docking having various scoring functions and cavity detection tools to reach a consensus about a potential binding site. The consensus technique is essential for large-scale screening because it enables a collection of programs and tools to function as a cohesive group and agree on the prediction, notwithstanding the robustness to failures and incorrect predictions of any one program. Therefore, the identification of binding sites is enhanced by a consensus on the predictions generated by molecular docking methods and cavity detection tools. Improved binding site identification ultimately enhances the performance of blind docking in terms of correctly identifying the binding mode of the ligand. Besides improved performance, we constructed an automated end-to-end pipeline to perform the docking of multiple ligands to multiple targets to enhance the practicality. The CoBDock pipeline is freely and publicly available for academic use: https://github.com/DavidMcDonald1993/cobdock.

## Methods

CoBDock automates the entire docking pipeline, from input preparation to binding site prediction through parallel blind docking and cavity-detection and, finally, executing local docking at those predicted binding sites for high-quality binding mode predictions. First, it prepares targets and ligands before executing blind docking using four molecular docking algorithms: AutoDock Vina [7], GalaxyDock3 [22], ZDOCK [23], and PLANTS [24]. In parallel, it identifies binding sites using two-cavity detection tools: P2Rank [15] and Fpocket [16]. To aggregate the predicted binding sites and modes identified by all the cavity detection and molecular docking algorithms, we drew a 10 Å-resolution grid over the entire protein and assigned each mode/binding site to the closest grid box. Finally, the grid locations were assigned and ranked by a machine learning (ML) predicted binding site score and the top-ranked location was selected. This location was mapped back to the closest cavity found by one of the cavity detection tools. Finally, to produce a final binding mode prediction for the ligand, we executed PLANTS [24] at the closest binding site.

**Fig. 1:**
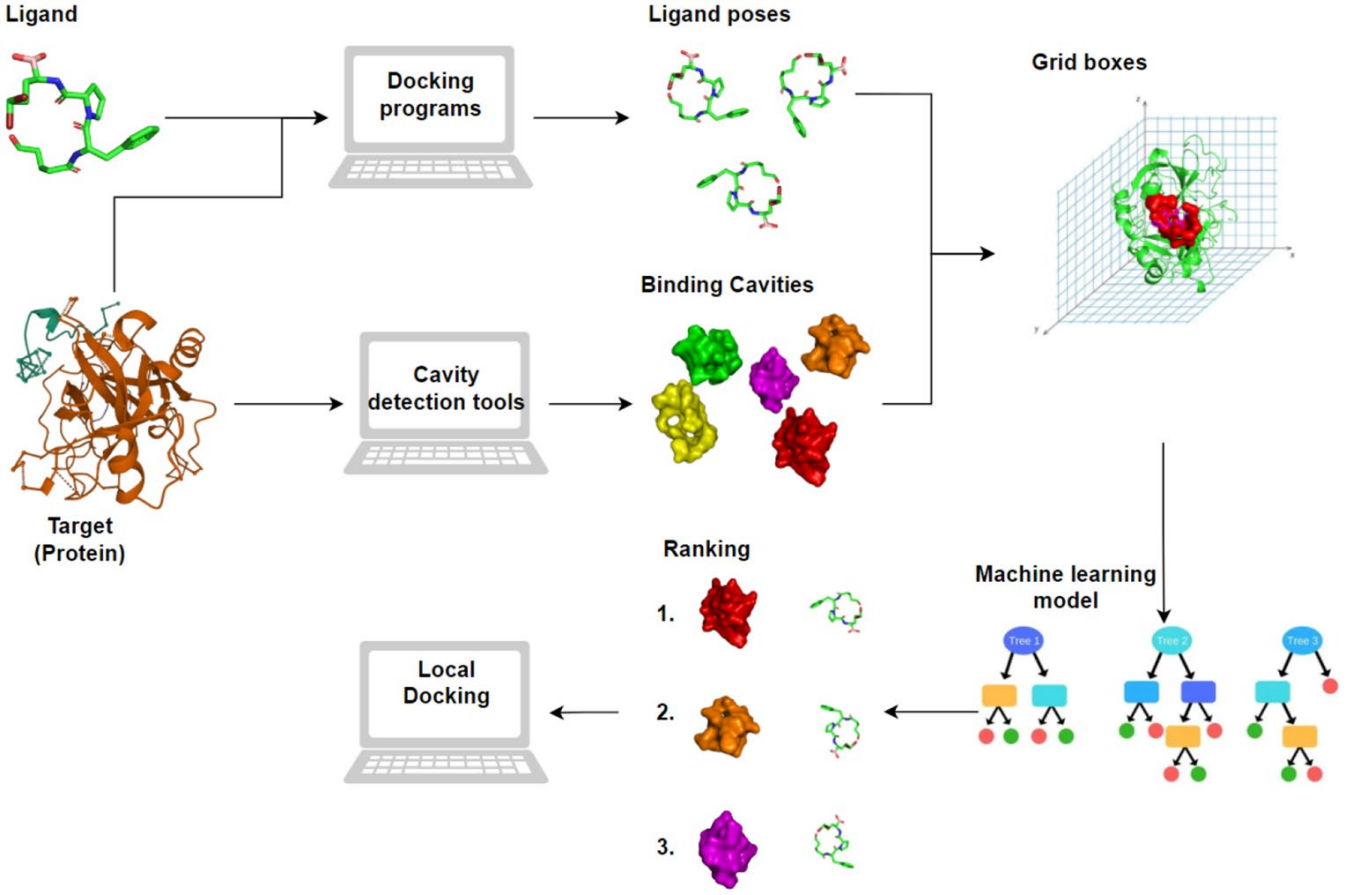
Schematic representation of CoBDock blind docking workflow. The docking methods, AutoDock Vina, PLANTS, GalaxyDock3 and ZDOCK, and binding site detection tools, P2Rank and Fpocket, are all executed by CoBDock in parallel. A three-dimensional 10 Å-resolution grid is drawn over the protein, and each predicted binding mode and pocket is assigned to the closest grid box. Boxes containing no binding modes or pockets are subsequently removed. Each remaining grid box is assigned an ML-computed “pocket score” that is used to rank them. The pocket closest to the top-ranked box is then selected as the true binding site. After binding site selection, molecular docking is executed at the binding site to produce the final binding mode for the ligand

The entire CoBDock pipeline consists of five steps (shown in Fig. 1): (1) docking methods, (2) cavity detection tools, (3) voxelization: Processing 3D structural data into grids, (4) using a trained machine learning model to score and rank voxels and (5) local docking to produce the final predicted binding mode of a ligand. The identification of binding sites and pose prediction performance were tested on PDBBind 2020, ADS, MTi, CASF-2016 and DUD-E and compared with common, Fpocket, and state-of-art studies, P2Rank and CB-Dock, achieving state-of-the-art results.

### **Docking methods**

CoBDock automates each step in Fig. 1, and provides faster and more practical docking steps to overcome difficulties when screening large target datasets.

### Target preparation

Users must provide a.pdb file or.pdb file list for a target as input. Also, a user can provide PDB IDs in a list or text file as input.

CoBDock cleans targets according to molecular docking protocols in its pipeline by removing undesired elements, including water, free ions, free atoms, and bound ligands by using Pymol [25]. In addition, CoBDock employs the Pdb2Pqr [26] software to provide protonation to the target molecules, specifically at a pH of 7.4. COBDock uses an AMBER [27] force field and propka [28] for titration states during protonation.

### Ligand preparation

Users must provide CoBDock with a ligand in the following formats: SMILES,.pdb,.mol,.mol2,.sdf (or multi files). CoBDock prepares them by adding hydrogen(s) to polar atoms using Open Babel with a preset physiological pH of 7.4 being utilized [29]. Finally, CoBDock uses OpenBabel [29] to convert them into the input format for each docking method.

### Blind docking

Blind docking is executed to search the entire protein to determine pose prediction. The utilization of multiple blind docking programs can yield diverse conformations situated at distinct spatial positions. Examining these diverse conformations can provide valuable insights for enhancing the effectiveness of the machine learning model in blind docking. Thus, the machine learning model generates predictions based on consensus on the various molecular docking outputs Fig. 1.

Consensus docking is a viable method to increase the performance of blind docking, which combines multiple molecular docking methods to provide higher performance [30, 31]. However, the performance of scoring functions depends on the dataset, so their performance ranges from 0–92%, depending on the benchmark under study [20]. To increase performance, we constructed a consensus docking pipeline, CoBDock.

Molecular docking methods have been used to identify the binding site, such as COACH-D [13], and GalaxySite [17]. Scoring functions find binding sites and ligand poses by searching the entire protein surface for favourable binding poses. We selected four representative molecular docking methods, Vina, PLANTS, GalaxyDock3 and ZDOCK, due to their relative advantages and different pose search approaches, see Table 1.

The scoring function utilized in Vina is an empirical scoring mechanism that draws significant inspiration from X-Score [32]. The efficiency of Vina in performing docking can be attributed to the absence of directional or theta-dependent terms. This characteristic enables Vina to build a triangular matrix at the beginning of the program, which comprises atom-pair evaluations within a distance cutoff of 8 Angstroms. The utilization of a matrix facilitates the examination of atom-pair interactions, hence expediting the docking process [33].

AutoDock Vina employs a united-atom scoring function, which exclusively considers the heavy atoms in the scoring process [7] to calculate the fitness or affinity of protein-ligand binding [34]. During the calculation, it derives advantages from a hydrophobic term, a non-directional hydrogen-bond term and a penalty associated with conformational entropy [35]. However, Vina has a deficiency in the treatment of electrostatic interactions and solvation effects, instead employing a potential function reminiscent of the van der Waals forces [35].

PLANTS is comprised of two score functions: PLANTSCHEMPLP and PLANTSPLP. Both are derived from previously reported scoring functions and force fields, primarily in terms of their functional form. The utilization of the piecewise linear potential (PLP) scoring function is employed in both scenarios to represent the steric complementarity between the protein and the ligand [36]. The PLANTSCHEMPLP score function incorporates the utilization of GOLD’s Chemscore implementation to provide angle-dependent terms for hydrogen bonding and metal binding [37]. In order to consider interactions, the combination of the torsional potential derived from the Tripos force field and a heavy-atom clash term is utilized [36].

The protein-ligand docking process in GalaxyDock2 incorporates a hybrid scoring system to enhance accuracy. The score can also be utilized in GalaxyDock2, a protein-ligand docking software that incorporates the conformational space annealing (CSA) algorithm, a global optimization strategy [38]. The CSA algorithm employs a population-based iterative optimization strategy to build a collection of low-energy conformations that have been locally reduced. The CSA population strictly adheres to specified mutual distances, which facilitates the concentration of conformational sampling on more profound minima during each iteration. The process of global optimization encompasses the manipulation of ring conformation and the reconstruction of the internal ligand structure, allowing for the adjustment of many degrees of freedom, such as bond angles and lengths. GalaxyDock3 has the same energy components as the GalaxyDock BP2 score with additional bonded energy terms to train its hybrid scoring function [22]. For example, bonded energy terms for ligands represent the bond angle, bond length, dihedral angle, and improper torsion angle energies of CGenFF [39]. The additional terms used to train the scoring function improved the performance [22].

ZDOCK has been purposefully developed to execute blind docking of protein-protein interactions. Hence, it exhibits inherent dissimilarities when compared to three other small molecule-protein docking programs, namely Vina, GalaxyDock3, and PLANTS. Understanding the distinctions and associations between ZDOCK and small molecule-protein docking programs could potentially provide valuable insights for the development of an effective machine-learning model. Additionally, the ZDock docking program demonstrates a notably diminished failure rate in comparison to PLANTS, Vina, and ZDock when executing blind docking procedures. The absence of missing data is essential for ensuring the strong functioning of our model. In addition to exhibiting a reduced failure rate, ZDOCK offers a multitude of ligand postures, numbering in the hundreds. The increased output quantity facilitates the detection of a wide range of ligand poses capable of occupying multiple binding sites on the protein. This capability facilitates comprehensive sampling of the complete protein structure.

The scoring function of ZDOCK incorporates three components, namely the IFACE Statistical Potential, Shape Complementarity, and Electrostatics, in order to enhance the docking performance. The term “IFACE statistical potential” is employed in the field of protein docking to characterize the interplay between pairs of amino acids situated at the interface of a protein complex. The calculation of the IFACE potential involves the utilization of a statistical potential that has been trained on a comprehensive database containing verified protein-protein interactions [40]. The concept of shape complementarity is employed in protein docking to characterize the extent to which the shapes of the protein and ligand exhibit mutual compatibility. When the protein and ligand exhibit compatible conformations, they can establish a more intimate binding, resulting in enhanced stability [41]. The concept of electrostatics is employed in the field of protein docking to elucidate the interplay between charged amino acids present on both the protein and the ligand. The stabilization of protein-ligand binding is reliant upon the significance of electrostatic interactions [42].

**Table 1:** The summary of molecular docking methods’ unique features and scoring functions, used in the CoBDock

Docking Methods	Pose research method	Advantages
Autodock Vina	An empirical scoring function that was largely inspired by x-score [33]	$\bullet$ High performance: 81% accuracy [43]
		$\bullet$ Fast
		$\bullet$ Ease of use
		$\bullet$ Most common
		$\bullet$ A high number of different pose locations
ZDOCK	Energy-based scoring function (IFACE Statistical Potential, Shape Complementarity, and Electrostatics)) [40]	$\bullet$ High performance: 85.71% [44]
		$\bullet$ Blind (Global) docking
		$\bullet$ A high number of poses
PLANTS	PLANTS(CHEMPLP) or PLANTS(PLP) derivied from piecewise linear potential (PLP) scoring function [36]	$\bullet$ High performance: 87% accuracy for the Astex Diverse Set (ADS) [24]
		$\bullet$ Relatively fast
		$\bullet$ A high number of variables related to ligand pose
GalaxyDock3	Global optimisation of a designed score function trained with an additional bonded energy term [22]	$\bullet$ High performance [23]
		$\bullet$ Ease of use
		$\bullet$ Flexibility
		$\bullet$ A high number of poses
		$\bullet$ A high number of different pose locations

The list of distinctive characteristics and scoring schemes for molecular docking technologies. An ideal scoring function is, in theory, the binding affinity determined by a thorough free energy simulation. However, using such a time-consuming method in docking investigations is not realistic. As a result, most scoring functions used today are based on force fields, empirical potentials, or knowledge-based potentials

Four molecular docking programs were selected, each with their default parameters, in order to ensure that any performance gain observed may be attributed only to the CoBDock application. Future work will investigate the impact of molecular docking parameter tuning on the entire CoBDock pipeline.

### **Cavity detection tools**

Conventional docking protocols require a binding site to perform docking. Two binding site methods are (i) experimental structure-based method that locates small native molecules on targets captured by co-crystal structures [45] and (ii) using cavity detection tools, such as P2Rank and Fpocket, to identify a binding site. Since experimentally determined bound ligands are not available for all targets, we omit (i) from the CoBDock pipeline and consider only tool-based predictions of cavities.

**Table 2:** The summary of cavity detection tools used in the CoBDcok

Cavity detection tool	Type	Overview
P2Rank 2.0	Machine learning	P2Rank is a standalone template-free program for machine learning-based prediction of ligand binding sites [15].
Fpocket	Geometric	A pocket detection tool called Fpocket was developed using alpha spheres and Voronoi tessellation [16].

The identification of binding sites has been a challenge in the field of structural research, prompting the development of several binding site methods throughout the years. A subset of individuals were provided with a brief introductory overview

P2Rank and Fpocket (Table 2) have been selected to develop our consensus blind docking program. The selected cavity detection tools, P2Rank and Fpocket, offer distinctive features that improve blind docking performance. P2Rank is a machine learning (ML) binding site detection tool that predicts the ligandability of nearby chemical neighbourhoods. P2Rank is a standalone application that can be integrated into automated pipelines. In addition, it offers high precision, rapid processing, and practicality.

We further incorporate Fpocket, the most prevalent cavity detection tool, to strengthen our blind docking infrastructure. Fpocket is a utility for pocket detection that utilizes Voronoi tessellation and alpha spheres. It is a simple, quick, and precise standalone tool that is suitable for an automated pipeline. These characteristics make them promising candidates for an automated pipeline, so we incorporated them into our CoBDock program [16].

### **Voxelization: processing 3D structural data into grids**

**Fig. 2:**
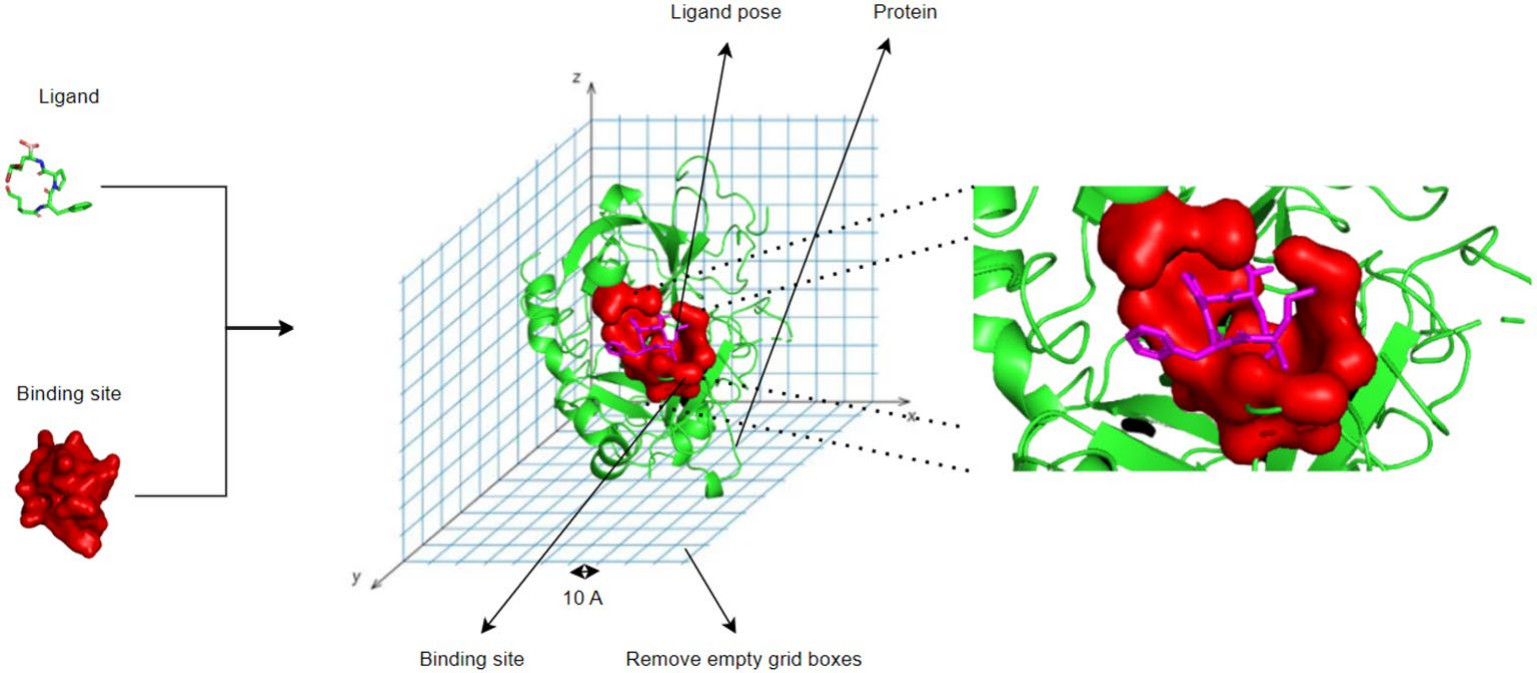
Representation of voxelisation processing for a protein (PDB ID: 1A3E) structure using PyMol. Ligand poses and binding cavities (site) are the outputs of molecular docking methods in magenta, and cavity detection tools in red and grid boxes convert these outputs into vectors. Empty grid boxes are filtered before using grid data to train a machine-learning model

CoBDock uses four molecular docking methods and two cavity detector tools to acquire ligand poses and cavities on a target protein. We used a voxelisation approach to convert 3D structural data to grids by fusing two sources of information. Each grid box comprises many channels that describe distinct forms of molecular docking and cavity detection tool results Fig. 2. The grid boxes train a classification model to rank voxels; then, the model makes a prediction to identify binding sites.

Voxels have been utilized for the purpose of sampling the complete protein structure. The results of programs become a feature of that voxel, once a voxel contains a found binding site and/or ligand poses. Besides the program features, the number of poses present in the voxel is determined in order to determine the frequency of poses, labelled as “sampled_pose_number_PROGRAM_NAME_at_location”. Also, we calculated the distance from the mass centre of the cavity/pose to the centre of the voxel, labelled “PROGRAM_NAME_distance”. As a result, the machine learning model has the capability to acquire knowledge regarding the frequency with which a program identifies the place, as well as the frequency with which programs identify the location as a binding site.

In voxel-based representation, the grid size is crucial for data processing to handle missing samples, increasing noise, or increasing redundancy. A large box size results in data loss, whereas a small box size results in computationally expensive procedures, noise, and redundancy. Large grids contain multiple results per grid, which can also result in data loss. In addition, small grid cells dramatically increase noise and redundancy, which makes the procedure computationally expensive. Therefore, we selected 10 Å from the literature to sample the entire protein structure [46].

### **Machine learning model to rank voxels**

A machine learning-based classification model assigns a binding site score between 0–1 to each voxel. The highest-scored voxel is mapped back to the closest cavity found by either Fpocket or P2Rank and this cavity is taken to be the predicted binding site of the ligand.

*Model training data preparation:* We used an EDock training set having 400 non-redundant proteins [18]. To determine 3D structure similarity across datasets, target structure pairs were formed by selecting one instance from the training set and one instance from each benchmark set. Subsequently, the TM scores were computed for the aforementioned couples [47]. In the case that the TM-score between a training and validation protein structure exceeded 0.5, the training protein structure was removed from the training set [47]. Hence, it is concluded that there exists no structural relationship between the train and benchmarks.

After removing proteins with more than 0.5 TM-Score, the rest of the 290 proteins in the EDock training set were used in molecular docking methods and cavity detector tools. Then, the outputs of the program were processed into 18,533 grid boxes. A box containing the co-crystalized ligand in the original target structure is assigned a true label. The other boxes are labelled as negative. Finally, missing data was replaced with the average feature, also known as mean value imputation.

The molecular docking and cavity detection methods exhibit missing values with a failure ratio ranging from 0 to 2% on the training set. Among the considered software applications, namely GalaxyDock, PLANTS, Vina, and Fpocket, it is seen that the former three exhibit a failure ratio of 2%, however, Fpocket demonstrates a comparatively lower failure ratio of 1%. Fortunately, both ZDOCK and P2rank do not exhibit any missing data. The failures observed in the blind docking of CoBDock can be attributed to the utilization of huge search sizes. The huge search size requires high memory usage. Performing blind docking necessitates significant computational resources due to the high level of flexibility and extensive search space involved.

CoBDock performs local docking to determine the final ligand pose. Fortunately, local docking focuses on specific locations to find ligand pose, therefore, each molecular docking has achieved a failure rate of zero on the training set.

**Fig. 3:**
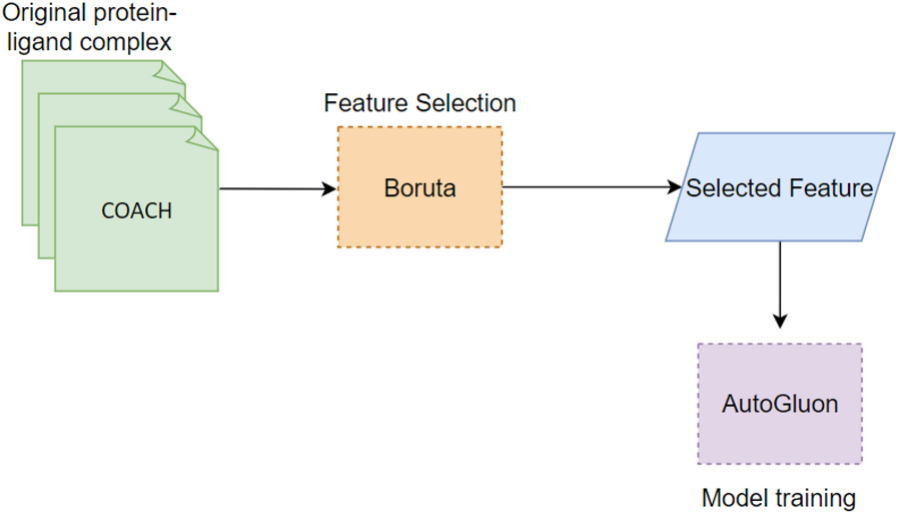
Schematic representation of the feature selection workflow and training model. The EDock training set is used in the Boruta package to select the most promising features. Then, selected features have been utilized to train a model using AutoGluon. AutoGluon automates splitting data, validation and stacking to train model

*Feature selection:* The training set was partitioned into two subsets, namely the training subset (80%) and the validation subset (20%), subsequent to the use of TM-score filtration. Feature selection was performed over the entire feature set of the training set. The optimum features reduce the complexity and overfitting of the model. Also, feature selection enhances the performance of the model. Therefore, we utilized the Boruta feature selection software to select the optimal features [48] (Fig. 3).

The Boruta algorithm [48] is a feature selection strategy utilized to identify the most significant attributes within a given dataset, with the aim of enhancing the performance of machine learning models. This technique achieves its objective by optimizing the amount of features included in the models. Boruta compares the relevance of each characteristic to “shadow” features – random permutations of the original features. Characteristics with consistently greater relevance than their shadow characteristics are retained, whereas elements with equivalent or lower importance are eliminated [48].

Boruta’s significance measure uses a tree-based classifier from Scikit-Learn (1.1.2) [49] to capture complicated feature-target variable correlations. Shadow characteristics assist Boruta in differentiating signals from noise, improving its feature selection process. This reduces overfitting, improves model generalization, and improves interpretability [48].

We analysed feature selection results on the validation set by using The ANOVA f-test Feature Importance, and Radviz Visualization. The ANOVA f-test Feature Importance, also known as Analysis of Variation, is a statistical technique employed to assess the value of selected features in elucidating the variation or disparities observed in the target variable within a given training set. Radviz, also known as Radial Visualization, is a data visualization approach that is employed to visually represent multivariate data within a two-dimensional spatial context. This approach is efficacious in comprehending the interrelationships and patterns among numerous variables concurrently. The Radviz plot employs a circular representation where each data point is depicted as a point positioned within the circle. The circular location of a data point is established by the equilibrium of pressures exerted by the variables, which act as attractive forces, drawing the points towards their individual places.

*Training machine learning model:* Autogluon version 0.8.0 has been used to train the voxel-scoring model in CoBDock [50]. AutoGluon is a library for automated machine learning (AutoML) that facilitates the training and deployment of machine learning models, even in the absence of previous experience in the field of machine learning. AutoGluon operates by automating the following tasks: (i) data preprocessing, and construction model architecture using bagging, multi-layer stacking ensembling techniques.

The process of data preprocessing is a crucial component in the preparation of data for modelling purposes. AutoGluon, a software program, facilitates the automation of data cleaning and transformation procedures, thereby guaranteeing the data is appropriately formatted for further analysis and modelling tasks. Also, Autogluon uses mean value imputation, a technique that involves the replacement of missing values with the arithmetic mean of the existing data. AutoGluon possesses the capability to autonomously identify and address missing values within a given dataset by employing mean value imputation. The proposed approach is a straightforward yet efficient imputation technique applicable to both numerical and categorical variables [50].

When the parameter auto_stacking is set to True, AutoGluon will employ bagging and multi-layer stack ensembling techniques in an automated manner to enhance the accuracy of predictions. This process involves the training of many models on distinct subsets of the data, followed by the aggregation of their predictions to generate a final forecast [50].

Bagging is a statistical technique employed to mitigate variation by training several models on distinct bootstrapped samples derived from the dataset. Bootstrapping is a statistical sampling strategy that involves the resampling of data with replacement. This implies that certain data points may be incorporated into many models, but other data points may not be incorporated into any models [50].

Stacking is a methodology employed in machine learning that involves the amalgamation of predictions generated by different models in order to derive a final prediction. The process involves training a meta-model using the predictions generated by the basis models. The meta-model acquires the ability to integrate the predictions generated by the basic models in order to enhance the accuracy of the final forecast [50].

### **Binding site prediction**

The output of the model is a predicted binding site score, ranging from 0 to 1 for all voxels. The final binding site prediction is given as the closest predicted cavity to the top-scoring voxel.

### **Ligand binding pose prediction**

CoBDock performs blind (global) docking using molecular docking to process 3D structures into voxels by searching entire proteins. However, the large search area in blind docking reduces the performance of ligand pose prediction [12]. Therefore, we preferred to use the local docking approach to improve the final pose prediction performance.

The voxel-scoring machine learning model in CoBDock orders grid boxes to identify the binding site. The grid boxes are utilized to identify the nearest pocket detected by either Fpocket or P2Rank. The centroid of the predicted ligand binding site serves as the focal point for the search region employed in conducting local docking. CoBDock conducts local docking specifically targeting the highest-ranking binding site to perform local docking. When a user desires to conduct more docking operations at various places, CoBDock has the capability to accommodate additional sites as per the user’s request.

The CoBDock software employs PLANTS as its default local docking program, as indicated by the supporting evidence presented in Additional file 1: Fig. S12, which reinforces the justification for this choice. Additionally, users have the option to convert the aforementioned docking process into Vina or GalaxyDock3, enabling them to perform local docking. In the event of PLANTS’ failure, CoBDock will resort to using Vina and subsequently GalaxyDock3 for the purpose of conducting local docking.

The CoBDock algorithm exclusively employs a 15A range inside specified coordinates of the first pocket for the purpose of conducting local docking, with default parameters. The presence of default parameters hinders the improvement of pose prediction performance due to the optimized docking conditions. Hence, the identification of the binding site by CoBDock is the sole factor contributing to performance enhancement.

### **Benchmarking binding site and binding pose prediction**

The three benchmarks utilized in this study were obtained from databases and a study: DUD-E, CASF-2016 and CB-Dock. As an additional benchmark, we sampled the latest version of PDBBind to sample updated PDB entries. We evaluated comparative pipeline performances using the five varied datasets below:

#### DUD-E

The Directory of Useful Decoys, Enhanced (DUD-E) is designed to assess the efficacy of docking programs and cavity detection tools. DUD-E incorporates proteins with diverse binding site characteristics, such as variable diameters, geometries, and electrostatic properties [51]. This variation assesses the ability of methods to accurately detect and predict binding sites across a broad range of circumstances. We used the DUD-E validation set, containing 102 X-ray structures of the targets from the DUD-E benchmark. Additionally, the DUD-E set has 26 kinases, 15 proteases, 11 nuclear receptors, 5 GPCRs, 2 ion channels, 2 cytochrome P450s, 36 other enzymes, and 5 miscellaneous proteins [18].

DUD-E, the docking tests exclusively utilize the active drug for each target, disregarding the decoy compounds. This is because decoy compounds lack a co-crystallized ligand, which is necessary for comparing against the expected conformation [18].

#### CASF-2016

The CASF-2016 dataset is composed of 285 protein-ligand complexes that possess high-quality crystal structures and dependable binding constants. The dataset consists of a collection of protein-ligand complexes characterized by high-quality crystal structures and dependable binding data. The approach used to determine the primary test set for CASF-2016 used the 4057 protein-ligand complexes contained in the PDBbind refined set (v.2016) [52].

The CASF benchmark offers measures for evaluating scoring functions across various activities. The newest CASF benchmark is CASF-2016. On this benchmark, over 30 classical scoring functions for pose prediction were evaluated. It has been used to evaluate scoring power, ranking power, and docking power against other significant scoring functions as a well-known benchmark [53]. Hence, we incorporated such a benchmark into our comparative analysis after removing protein having higher than 0.5 TM-score according to our training set. Finally, we had 266 proteins in the benchmark (Additional file 1: Fig. S11).

#### Astex diverse set (ADS)

A well-known benchmark dataset for measuring the effectiveness of cavity identification tools and docking systems in the field of structure-based drug discovery is the Astex Diverse Set (ADS) [54]. It comprises a variety of drug-like ligands and relevant therapeutic targets. In assessing docking algorithms or cavity identification methods, the ADS is crucial for a number of reasons, including (i) diversity of ligands, (ii) diversity of binding modes, (iii) a standardized benchmark, (iv) realistic drug design cases, (v) a well-established benchmark [14].

#### MTiOpenScreen set (MTi)

The test data used in this study were obtained from the benchmark set of MTiOpenScreen [55]. MTi also includes a variety of 27 different crystal structures of important pharmacological targets, such as nuclear receptors, G Protein-Coupled Receptors (GPCRs), and enzymes. Therefore, it is a good test of the accuracy and robustness of docking programs across different target classes [14]. In accordance with the CB-Dock procedure, we used a set of 27 complexes gathered by them in our study as a baseline for evaluation. Utilizing MTi as a benchmark has the potential to mitigate the inherent bias in comparative analysis.

#### PDBBind (General set)

PDBbind is extensively utilized within the community of computational drug design, making it a benchmarking standard [56]. The PDBbind database contains a diverse collection of protein-ligand complexes, spanning a broad spectrum of target proteins, ligand sizes, and binding modes. In addition to the aforementioned characteristics of the general set, the PDBBind general set has been employed to depict the efficacy of research involving low-quality data [57, 58]. The absence of a high-quality PDB file for a target protein might be considered a practical challenge. In such cases, the utilization of a low-quality benchmark can serve as a valuable tool for evaluating the reliability and effectiveness of computational pipelines. Furthermore, the utilization of subpar benchmarks may be employed to subject models to rigorous stress testing. If a model has strong performance on a benchmark of poor quality, it implies that the model exhibits more robustness and less susceptibility to noise [59]. Therefore, we selected the most updated PDBBind v2020-general set to represent low-quality data.

The entire automated blind docking programs, such as CB-Dock, provide their protocol as a web server to execute one by one pair. Therefore, using the entire PDBbind as a benchmark is time-consuming, so we randomly sampled 522 protein-target complexes from the PDBBind v2020-general set. Then, the TM-score was calculated pairwise in 522 to remove structural overlap with the other three benchmarks. To eliminate proteins with similar structures in the PDBBind general set, we computed the pairwise TM score and removed proteins with TM values over 0.5. The TM scores for proteins in the PDBBind general set have been computed for all benchmarks as a second step. Once a protein’s TM score exceeds 0.5, it is deemed unfit for further consideration and is subsequently rejected. The next step is the remaining protein molecules were subjected to a comparative analysis against the training set and all proteins with a TM-score greater than 0.5 with any structure in the training set were removed. In conclusion, a total of 53 proteins with a TM-Score below 0.5 were identified, showcasing significant diversity. [60].

While DUD-E primarily emphasises the inclusion of active, inactive, and decoy compounds to assess the screening capability of a docking program in distinguishing active compounds from inactive ones, the PDBbind dataset primarily focuses on predicting the binding pose of protein-ligand complexes with known binding geometry. Alt Additionally, the PDBbind dataset also evaluates the ranking ability of different compounds and the prediction of absolute affinity values. The presence of a diversified and well-designed benchmark is crucial for evaluating the performance of molecular docking systems. Therefore, we are validating our blind docking software based on binding site identification and posture prediction utilizing DUD-E and CASF-2016 as our core datasets.

Astex Diverse Set (ADS), and MTiOpenScreen Set (MTi) have been used in CBDock validation analysis, so we used them to reduce bias in our study by following CBDock [14].

The study incorporated the latest version of PDBBind (General Set) to examine the robustness of the pipeline and accurately represent data of inferior quality. As a result, CoBDock has undergone testing on notable benchmarks, namely DUDE and CASF-2016, which are recognized for their size and prominence. Additionally, three supplementary benchmarks have been utilized to provide further insight into the capabilities of CoBDock.

### **Comparison with state-of-the-art methods**

CoBDock contains two cavity detection tools, Fpocket and P2Rank, used to determine the binding site. Besides the unique features of Fpocket and P2Rank discussed above, they have significant cavity identification performance, especially P2Rank. P2Rank surpasses a number of currently available tools, such as two commonly used standalone programs (Fpocket and SiteHound), a thorough consensus-based tool (MetaPocket 2.0) (Additional file 1: Table S3), and a recent deep learning-based method (DeepSite). Therefore, CoBDock has been compared with Fpocket and P2Rank on binding side identification performance. In addition, subsequent to the discovery of binding sites by the utilization of Fpocket and P2rank, the obtained coordinates were employed to conduct local docking via our designated molecular docking program. A comparative analysis has been conducted to assess the efficacy of ligand pose prediction in the P2rank and Fpocket pipelines, in comparison to the CoBDock methodology.

CB-Dock is a recent protein-ligand docking tool that uses a blind docking approach to predict the binding poses of ligands to proteins after identification of the binding site. CB-Dock uses its designed cavity detection approach, called CurPocket. CurPocket is a computational approach utilized for the prediction of protein-ligand binding sites [61]. This method employs the calculation of curvature factors to identify and locate cavities present on the surface of the protein. In order to represent the blind docking pipeline, we used CB-Dock and CB-Dock2, the updated version, for comparison.

CB-Dock2 contains structural- and template-based pipelines. The process of template-based docking commences by utilizing a pre-existing structure as a reference point, establishing an initial foundation for the subsequent docking computations. The implementation of this approach has the potential to decrease the number of feasible conformations that necessitate exploration, hence enhancing the efficiency of docking computations. Hence, template-based docking is the easiest approach for docking [62, 63]. However, other pipelines perform blind docking (“free docking”) without pre-existing structures for a target or a ligand [63]. Hence, the present study exclusively evaluates CB-Dock2’s structural-based predictions for comparative analysis. Consequently, each pipeline employed in this research abstains from utilizing any pre-existing data, thereby mitigating potential biases.

In summary, CoB-Dock was compared against four different pipelines: Fpocket, P2Rank, CB-Dock and CB-Dock2 (From this point, the structural-based docking tool in CB-Dock2 shall be referred to as CB-Dock2.) on two different tasks: (i) binding site identification and (ii) ligand binding pose prediction.

### **Performance metrics**

#### Cavity identification accuracy

An 8 Å distance threshold from computational to experimental Ligand binding sites (LBSs) is the standard accuracy metric in docking [18]. Besides accuracy for each model, We calculated the mean and median distances between ligand binding sites (LBSs) predicted by the cavity detection tool and LBSs of the native structure to demonstrate cavity identification performance for each program.

#### Binding pose prediction accuracy

The root-mean-square deviation of atomic positions, or RMSD, is a measure of the average separation between the atoms of stacked proteins (typically the backbone atoms). The docking pose performance is evaluated by RMSD metrics between the predicted ligand docking pose and the native structure:

$$\begin{aligned} RMSD= \sqrt{\sum _{n=1}^{N} [(x_i-x_{i,ref})^2 + (x_i-x_{i,ref})^2 + (x_i-x_{i,ref})^2)]/N} \end{aligned}$$

where, are, respectively, the coordinates of heavy atom $i$ in the predicted and experimental model of the ligand. $N$ is the total number of heavy atoms. The tool obrms is used to calculate the symmetric RMSD between each ground truth and predicted ligand pose [29]. If the RMSD value is lower than 2 Å, the predicted ligand pose can be labelled as a true prediction; otherwise, it should be labelled as a false prediction to calculate accuracy [14, 18].

## Results and discussion

The unique feature of CoBDock compared with other blind docking pipelines is its data processing 3D structure into grid boxes using a voxelisation process. The data processing method allows a more interpretable machine learning model to be rapidly built compared to more complex deep learning models. Also, once the component numbers of molecular docking and cavity identification within the CoBDock pipeline are increased using our parallel process approaches, the binding site identification and pose prediction performance will be enhanced. Even combining six components into the pipeline to build the current version of CoBDock has great potential in blind docking because of the consensus approach. Hence, CoBDock has undergone rigorous testing, comparative analysis with contemporary models, and thorough examination.

As illustrated in Fig. 1, the intuition behind the CoBDock method is to integrate various docking and cavity detection methods in a hybrid parallel pipeline to increase the accuracy of identification of the binding sites and pose prediction in a blind docking setting. Each program and tool in the CoBDock individually search the entire surface of a protein, and the results can reach a consensus on binding location. Therefore, combining molecular docking methods and cavity detector tools results in robust docking performance.

The main metrics to evaluate the performance of the cavity-detected docking methods are identification of the binding site and pose prediction [14, 18]. Therefore, these assumptions were tested in two sections: (i) identification of the binding site and (ii) binding pose prediction.

### **Identification of binding site**

**Fig. 4:**
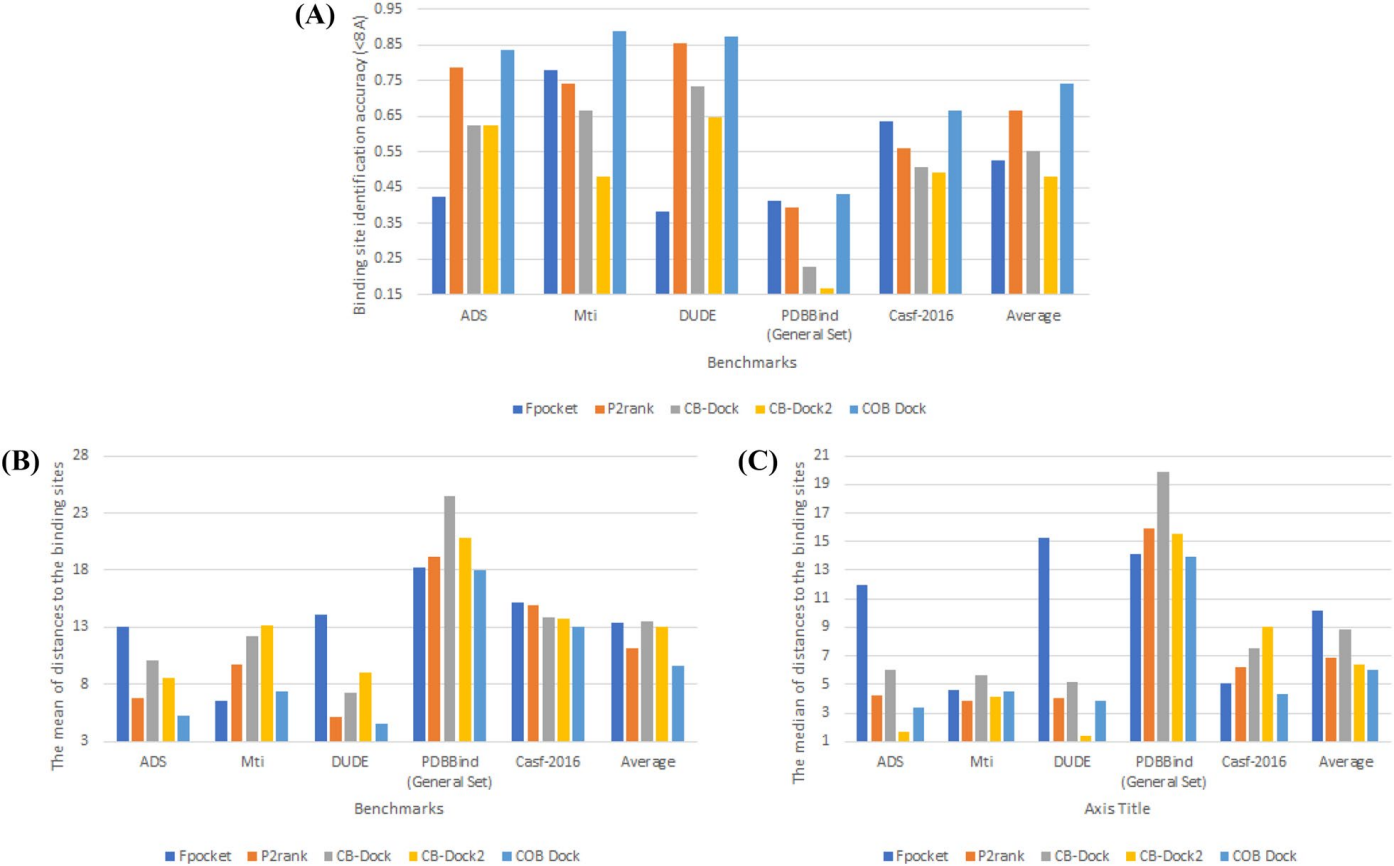
The binding site-prediction accuracy of CoBDock compared with state-of-art methods. The binding site-prediction accuracy of CoBDock was compared with four representative pocket identification algorithms (Fpocket, P2Rank, CB-Dock and CB-Dock1). The mean distance is the mean distance between the centroid of the ground true binding site and the centroid of the predicted binding site. Likewise, median distance is the median distance between true and predicted pocket centroids, better accounting for outliers. Accuracy (within 8 Å) is the proportion of predicted binding sites whose centroid was within a threshold of 8 Å of the centroid of the true binding site

The performance of the identification of the binding site directly affects the pose prediction performance in blind docking. Finding the correct ligand pose is only possible with the actual binding site or a good prediction of the binding site. Therefore, binding site identification performance is vital for any automated docking methods. One of the metrics for binding site identification performance is the distance between the pocket and the centroid of the native ligand. We used 8 Å to calculate the accuracy in Fig. 4 [18]. Additional metrics for evaluating the identification of binding sites include the mean and median distance from the centroid of the actual binding site. A decrease in both the mean and median values suggests that the predicted location is in closer proximity to the actual ground truth position. To quantify the overall performance of programs, the average metrics across all the ligand-target pairs in each dataset have been computed, and represented in Fig. 4 with an “Average” label.

The results of Fpocket, as a well-known cavity detector, show that the choice of the benchmark can drastically reduce accuracy from 0.78 to 0.38. Despite the considerable variability in the accuracy of Fpocket, which diminishes its reliability, it is noteworthy that Fpocket achieved the second-highest accuracy score of 0.635 among the programs evaluated in the CASF-2016 assessment. In contrast, CoBDock had an accuracy of 0.667 with minimal variability in its accuracy measurements. Fpocket demonstrated a modest level of competitiveness on the PDBbind benchmark, with an accuracy of 0.415. In comparison, our performance surpassed this with a higher accuracy of 0.434. CoBDock demonstrates a lower mean and median value (Fig. 4B, C) on PDBbind compared to Fpocket. The lower mean and median distance represent how close the predicted location is to the ground truth binding site. Therefore, it is plausible that the utilization of CoBDock might potentially enhance the RMSD performance when evaluated against the PDBBind benchmark.

P2Rank, as a machine learning cavity detector, is competitive against CoBDock on DUD-E. P2Rank provided 0.853 accuracy, while The accuracy of CoBDock was 0.872. Also, the mean distance of CoBDock on the DUD-E benchmark is 4.608, while its median distance is 3.867 Fig. 4. In comparison, P2Rank has a mean distance of 5.178 and a median distance of 4.086 on the same benchmark. Regarding the outcomes of CASF-2016, it was observed that CoBDock had a 10% enhancement in accuracy compared to P2rank, while also demonstrating lower mean and median values. These results suggest that the predicted position of CoBDock is closer to the ground truth than the anticipated location of P2Rank. Our method outperformed P2Rank on the other benchmarks, including ADS, MTi and PDBbind by providing 4–15% more accuracy. Also, CoBDock provided lower mean and median on these benchmarks 4B and 4C.

The blind docking pipelines, CB-Dock and CB-Dock2, were evaluated and compared to CoBDock on five benchmark datasets. The range of increase in accuracy for binding site detection versus CB-Dock pipelines ranges from 13 to 40%. The CoBDock pipelines exhibited significantly lower mean and median values compared to the CB-Dock pipelines across five benchmark datasets.

To demonstrate the overall performances of programs, we calculated the mean of metrics. The superiority of CoBDock in identifying the binding site compared to four other programs (Fpocket, P2Rank, and CB-Dock pipelines) is evident based on its higher average accuracy, as well as its lower average mean and median values Fig. 4.

### **Pose prediction**

We constructed a separate pipeline using Fpocket and P2Rank to identify binding sites. In order to assess the pose prediction performance of the two cavity detection programs, Fpocket and P2Rank, we execute PLANTS with a 15Ax15Ax15A about the centroid of the top-ranked pocket for each program. Finally, only the pose having the lowest energy on the top binding site is considered to calculate RMSD.

**Fig. 5:**
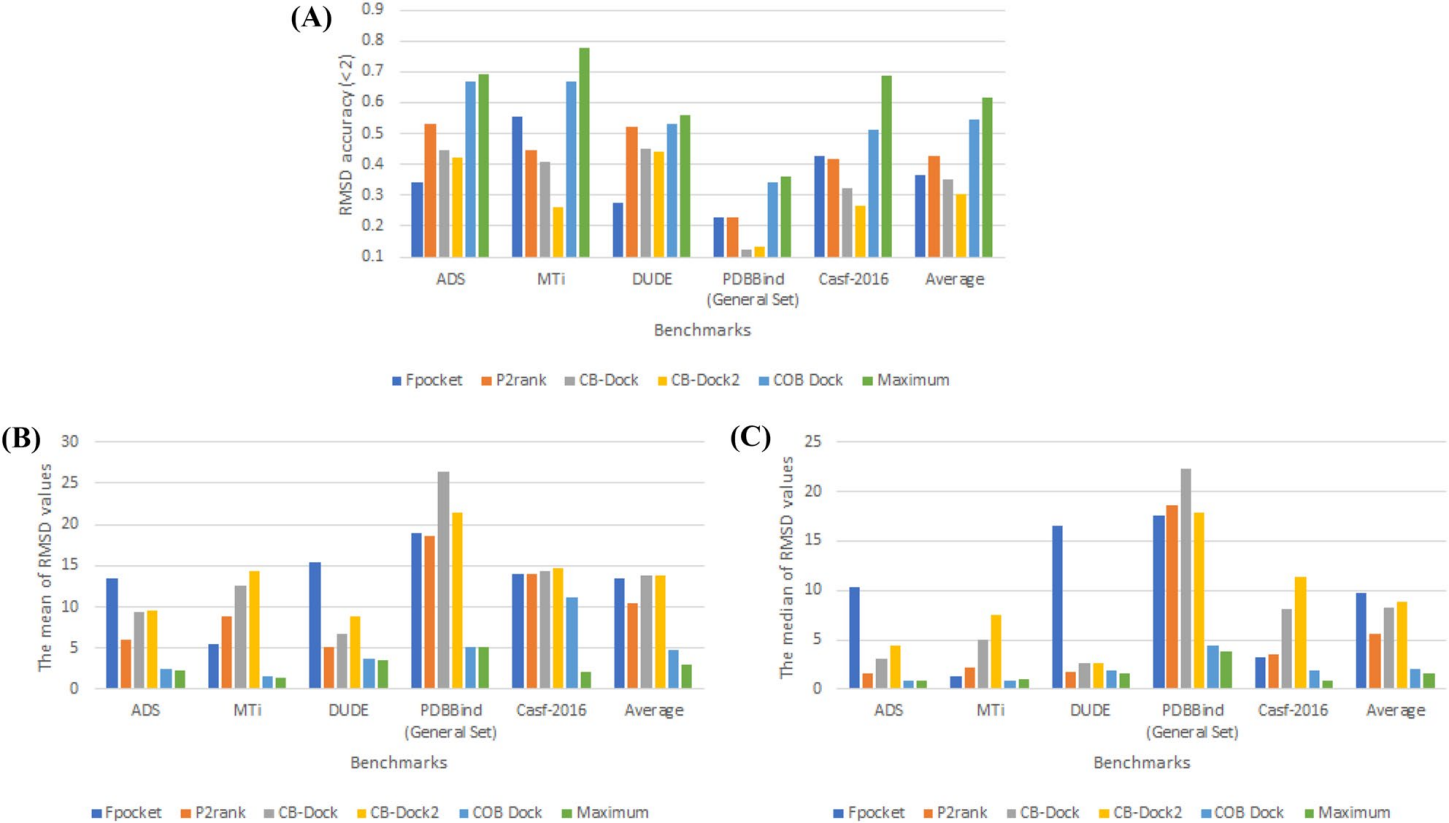
The pose prediction performance of CoBDock compared with state-of-art algorithms. The pose prediction performance of CoBDock compared with Fpocket, P2Rank, and CB-Dock (CB-0), structure-based blind docking of CB-Dock2 (CB-1), and template-based blind docking of CB-Dock2 (CB-2) on five benchmark datasets. The performance metric is mean and median RMSD. Mean RMSD is the mean RMSD between predicted ligand poses and true ligand poses. Likewise, median RMSD is the median RMSD between predicted ligand poses and true ligand poses. The accuracy is the proportion of predicted ligand poses whose poses were within a threshold of 2 Å of RMSD

CoBDock has shown superior performance compared to Fpocket, with an increase in accuracy ranging from 11 to 33% Fig. 5. The lower mean and median, shown in Fig. 5B, C, respectively, demonstrate that CoBDock consistently outperforms Fpocket in terms of pose prediction performance across all benchmarks.

P2Rank exhibited a competitive level of performance in terms of binding site prediction when evaluated against CoBDock using the DUD-E benchmark dataset. While CoBDock exhibits a slightly greater accuracy and lower median performance compared to P2Rank on DUD-E, it is noteworthy that CoBDock has a substantially lower mean RMSD, as seen in Fig. 5B. The significantly lower means indicate that CoBDock generally provides lower RMSD for protein in DUD-E. The study’s analysis of CASF-2016, which serves as a significant reference point, clearly indicated that CoBDock had superior performance compared to P2rank, with a 10% improvement in the accuracy of RMSD measurements. As for the other three benchmarks, it was observed that CoBDock exhibited superior results compared to P2Rank, with a notable increase in RMSD accuracy ranging from 13 to 22%. Additionally, the lower mean and median values seen in Fig. 5B, C provide evidence that CoBDock has superior performance in predicting ligands compared to P2Rank on ADS, MTi, and PDBbind.

As for CB-Dock pipelines, CoBDock exhibits superior performance compared to CB-Dock pipelines, with an increase in accuracy ranging from 8 to 44% across all benchmarks. The superior accuracy of CoBDock was substantiated by its lower mean and median values compared to the CB-Dock pipelines Fig. 5B, C.

The substantial under-performance of both CB-Dock pipelines in contrast to CoBDock can be attributed to two primary factors: (i) limited binding site identification performance and (ii) local docking parameters. The limited binding site performance of the subject under investigation is depicted in Figure 4. This limitation has a direct impact on the overall efficacy of local docking. The results presented in Figure 12 indicate a notable improvement in the accuracy of CB-Dock pose prediction by approximately 10% when PLATNS was employed as opposed to Vina. In order to enhance the performance of CB-Dock, it performs docking at five distinct cavities and thereafter reevaluates them based on their binding energy. This approach reveals that the cavity detection approach employed by CB-Dock exhibits certain limitations in accurately identifying binding sites. However, the utilization of solely the first cavity by CoBDock to achieve notable performance serves as compelling evidence of the enhanced predictive capabilities in binding site determination.

The average metrics in Fig. 5 indicate the overall performance of the root mean square deviation (RMSD). The CoBDock pipeline has an average accuracy of 0.567 across five benchmarks, whereas the P2Rank pipeline demonstrates an average accuracy of 0.441, as the second most successful pipeline. Additionally, it should be noted that CoBDock had the lowest mean and median values when considering the results of the five benchmarks. As a result, it is evident from Fig. 5 that the performance of CoBDock surpasses that of other pipelines without any ambiguity.

The ligand coordinates obtained from the ground truth have been utilized for conducting local docking, as depicted in Fig. 5, labelled as “Maximum”. The low accuracy difference between “Maximum” and CoBDock serves as more evidence supporting the efficacy of the CoBDock pipeline. Furthermore, the accuracies of “Maximum” indicate that there is potential for further enhancement in the optimization of local docking parameters or the implementation of consensus local docking methods (Fig. 5).

### **Ablation analysis**

Gaining a comprehensive understanding of the functioning principles of CoBDock can significantly enhance one’s comprehension of the process of identifying binding sites. Gaining a comprehensive understanding of the characteristics shown by the binding site can facilitate the development of a more precise pipeline for blind docking. Hence, before doing the case study, we examine the concepts of ablation and feature analysis.

The Boruta feature selection approach is employed to choose several characteristics from the output of each program in order to optimize performance. To determine the need for each component in CoBDock, an ablation analysis is conducted by systematically deleting each component individually.

**Fig. 6:**
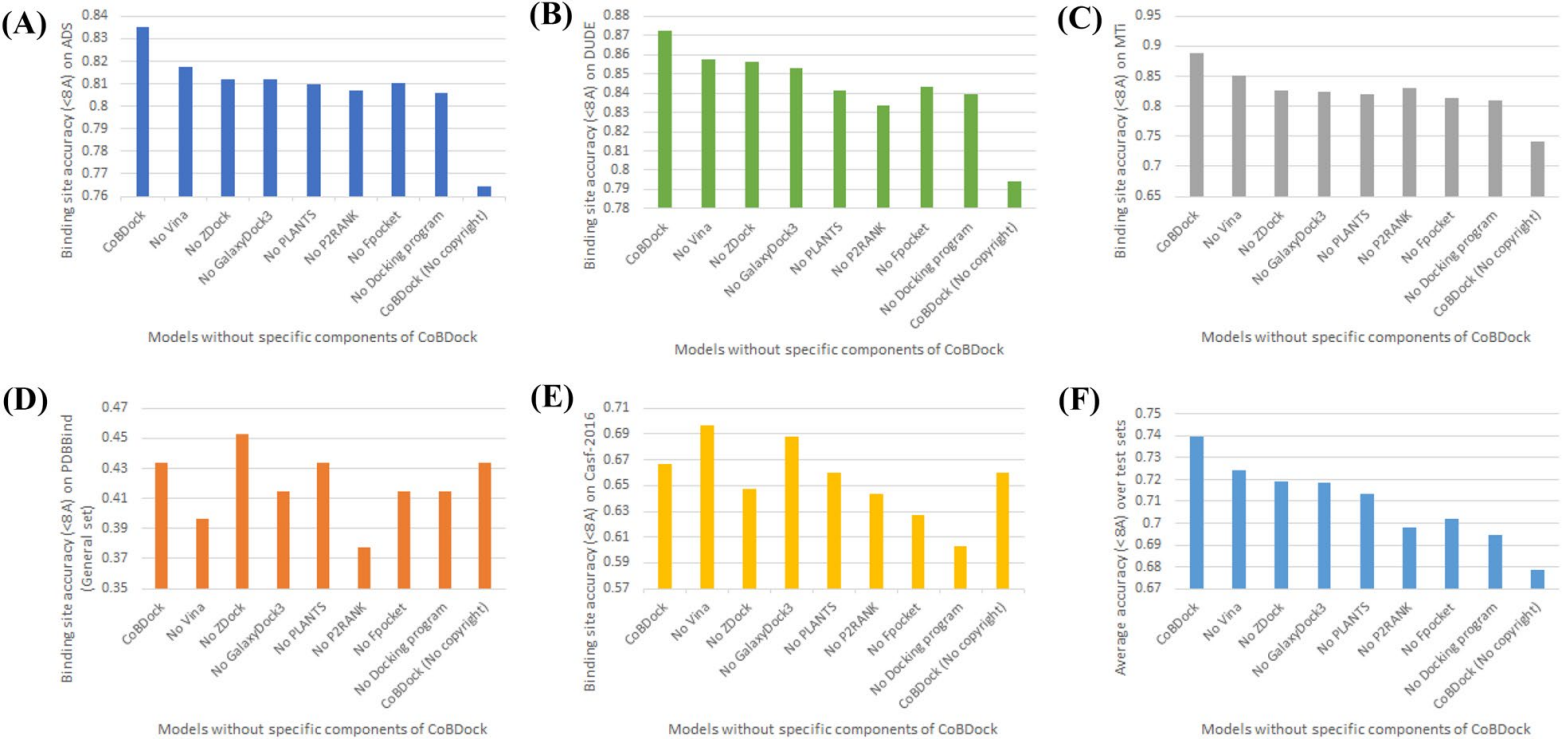
The summary of the ablation analysis conducted on five benchmark datasets. The bar charts labelled **A**–**D** depict the model’s performance on ADS, DUD-E, CASF-2016, PDBbind, and MTi, respectively. The first bars depicted in each bar chart illustrate the comprehensive performance of CoBDock. The remaining bars have been categorized based on the absence of a specific component. Furthermore, we have eliminated all cavity detection tools and molecular docking programs, which are depicted as the last bars on each bar chart

Using molecular docking methods as a cavity detection tool is an approach to identifying binding sites [13, 17, 64]. We designed to demonstrate that using our grid box sampling approach with docking methods can be competitive against a cavity detection tool specifically designed to order the cavity detection outputs. Although the utilization of just docking features during model training (as depicted by ’No cavity detection tool’ in Fig. 6) did not exceed the performance attained by exclusively using the cavity detection tool program model (as indicated by ’No docking programs’ in Fig. 6), it did give superior outcomes in comparison to Fpocket across all benchmarks, except PDBbind. ’No docking programs’ was 4–45% more accuracy than Fpocket on three benchmarks. Furthermore, it exhibited superior performance compared to P2Rank, with an improvement ranging from 1 to 7% across all benchmarks.

The performance of CoBDock was seen to decline when a component was eliminated from the pipeline in all benchmark tests, except PDBbind, providing compelling evidence that CoBDock requires all feature programs to enhance overall performance. The observed decrease in performance upon removing a single component suggests that CoBDock might potentially enhance its performance with the addition of more components. However, we leave an investigation into this as future work.

### **Feature analysis**

The Boruta feature selection method has been employed to identify the most promising features, hence enhancing the interpretability of the model. Gaining an understanding of feature significance is crucial in comprehending the predictive capabilities of CoBDock.

**Fig. 7:**
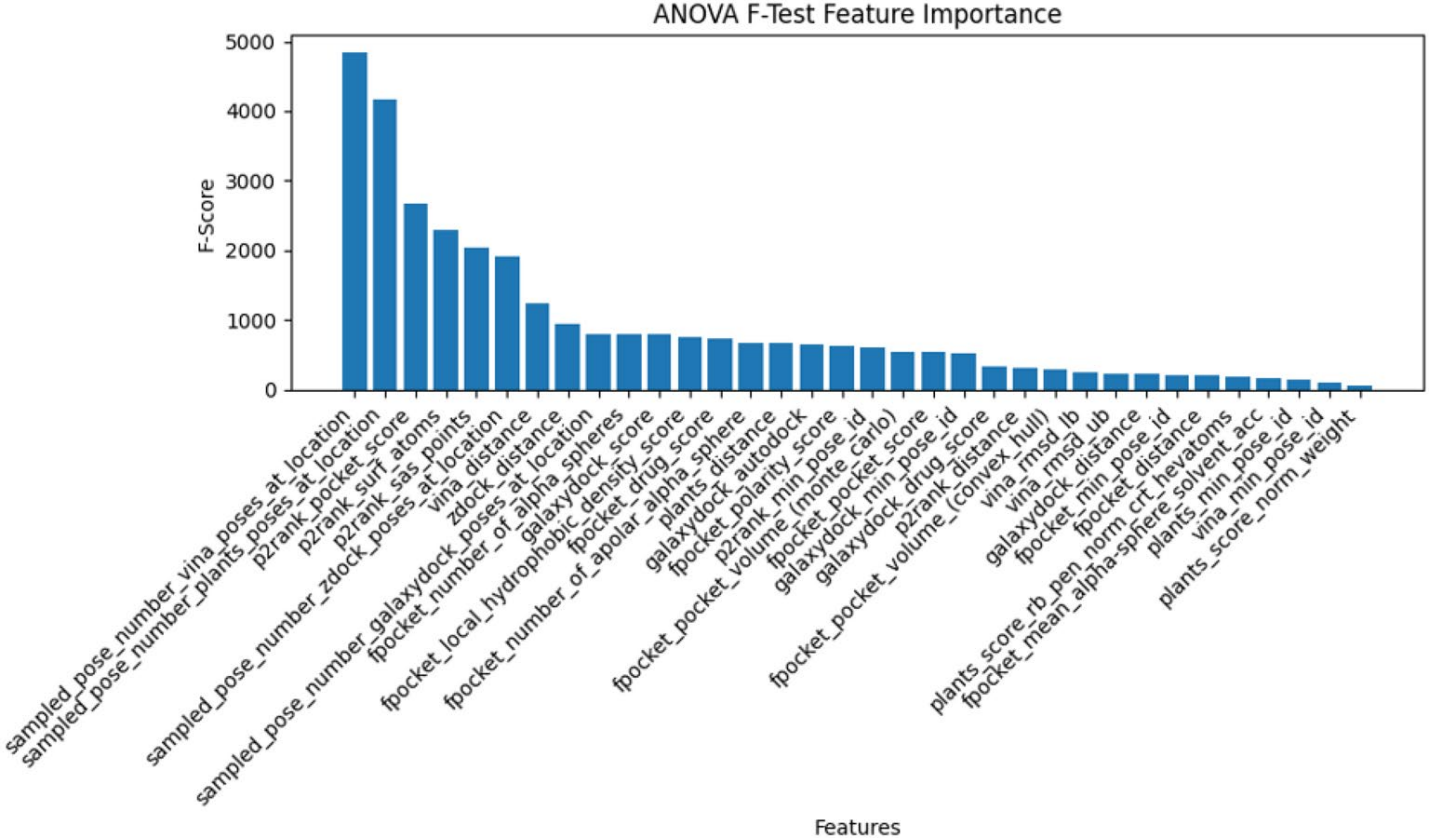
The feature importance for selected features by Boruta. Boruta selected features by optimising feature numbers. The selected features have been assessed using the ANOVA F-Test feature importance test. While the Y-axis demonstrates the F-score calculated by ANOVA, on the X-axis, the feature names in the format “PROGRAM_NAME”_“FEATURE_NAME” are displayed

Figure 7 demonstrates that the number of Vina and PLANTS poses number in voxel features has the the maximum F-scores. In addition, the number of ligand configurations in a voxel for GalaxyDock3 and ZDOCK has a relatively high importance score, placing them among the ten most essential features. We also computed the distance between the mass centre of ligand poses and the voxel centre, which we labelled “X_distance” (where “X” is the name of a component program). Two of them, vina_distance and zdock_distance, are also among the top 10 essential features of Fig. 7. Other docking-based features, such as GalaxyDock_drug_score, have been selected by Boruta, but they possess a modest level of significance.

Boruta’s feature selection retained the majority of cavity detection tool features for both Fpocket and P2Rank. In general, P2Rank features are more informative than Fpocket, supported by the P2Rank paper [15].

**Fig. 8:**
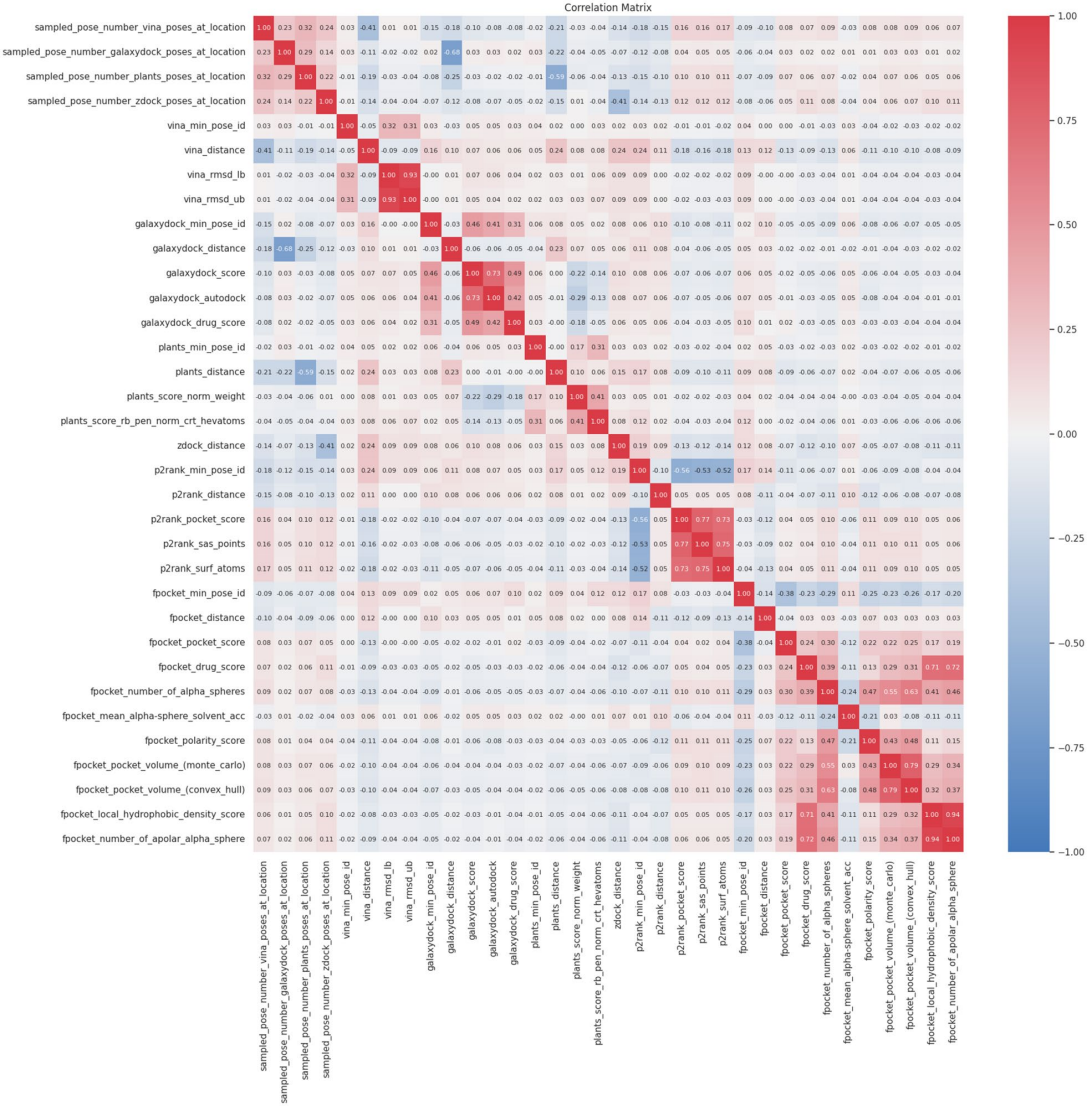
The heatmap to represent correlation score between selected features by Boruta. The correlation score is computed for every chosen pair of features. Blue (-1) indicates a strong negative connection between characteristics, whereas Red (+1) signifies a significant positive association. The colour white, denoted by the value of 0, signifies the absence of any association between the characteristics. The feature names are represented in the “PROGRAM_NAME”_“FEATURE_NAME” format

Figure 8 demonstrates a correlation between features selected by Boruta. Some of Fpocket features, such as Fpocket_local_hydrophobic_density_score and Fpocket_number_of_alpha_spehere, exhibited a strong positive correlation, resulting in the red colouration of the matrix’s terminal region. With the exception of the Fpocket association with itself, there is a lack of significant positive or negative correlations seen across the feature sets. The absence of a significant association provides evidence that our selection of molecular docking and cavity detection tools is sufficiently diversified to enhance the accuracy of binding site identification.

**Fig. 9:**
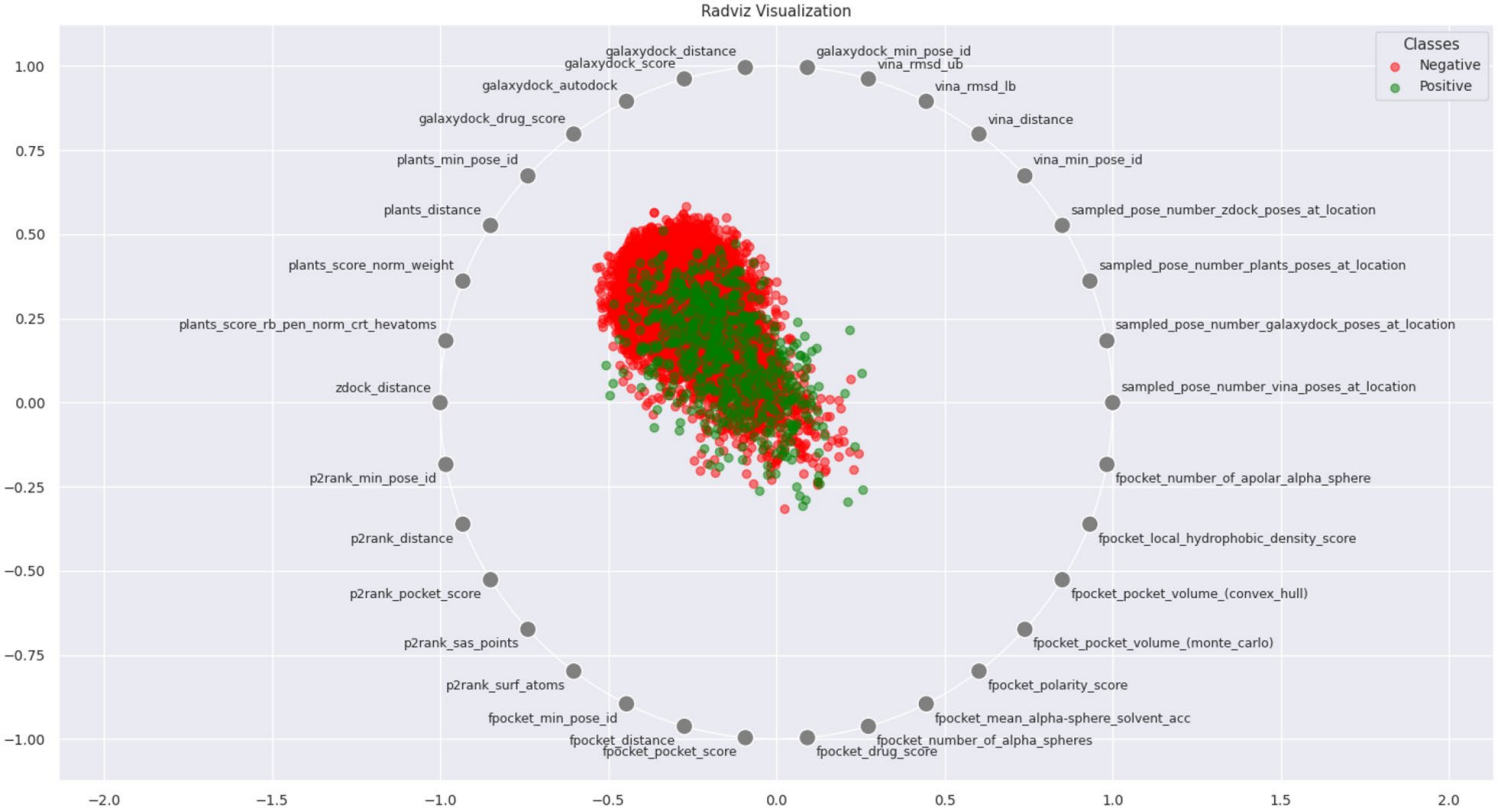
The representation of Radviz visualization for selected features by Boruta. The x and y coordinates of the data points correspond to the projected locations of the data points inside a two-dimensional space. It facilitates the identification of data clusters, patterns, and trends. Also, when a data point is situated at the origin (0, 0, 0), it signifies that the contributions of the characteristics (variables) represented by the axes are comparatively equitable and impartial for that specific data point. The feature names are represented in the “PROGRAM_NAME”_“FEATURE_NAME” format

The findings depicted in Fig. 9 demonstrate that both PLANTS and GalaxyDock3 display a discernible tendency to attract data points toward their respective positions within the Radviz visualization. The observed trend clearly indicates that the molecular docking algorithms exhibit a notable proficiency in accurately categorizing the binding location. The substantial impact exerted by these programs on the data points suggests that they make a substantial contribution to the differentiation and characterization of the binding interactions.

P2Rank was chosen as a competitive cavity detection tool due to its demonstrated high performance. However, it did not exhibit the behaviour of pulling data points towards itself in the Radviz visualization. However, the P2Rank_min_pose_id, which is positioned close to -1 on the X-axis, indicates a negative association between the pose id and the binding site. Specifically, a lower pose identification has a higher likelihood of accurately determining the binding point. Furthermore, it can be observed that the Fpocket_min_pose_id exhibits proximity to $-$0.5, indicating a comparatively lower potential in comparison to P2Rank in terms of its ability to accurately identify the binding site.

It is noteworthy that the ZDOCK_distance feature is situated in close proximity to P2Rank on the display, despite its distinction as a non-small molecule-protein docking tool. Furthermore, the negative value of ZDOCK_distance (-1) on the X-axis suggests that a lower ZDOCK_distance might potentially aid in the determination of the binding site. The variable “sampled_pose_number_zdock_at_location” denotes the numerical identifier assigned to a certain pose inside the grid box. The location of the feature, approximately at +1 on the X-axis, further suggests that ZDOCK should explore more poses inside the ground truth binding site.

### **Exploring the application of CoBDock: a case study**

**Fig. 10:**
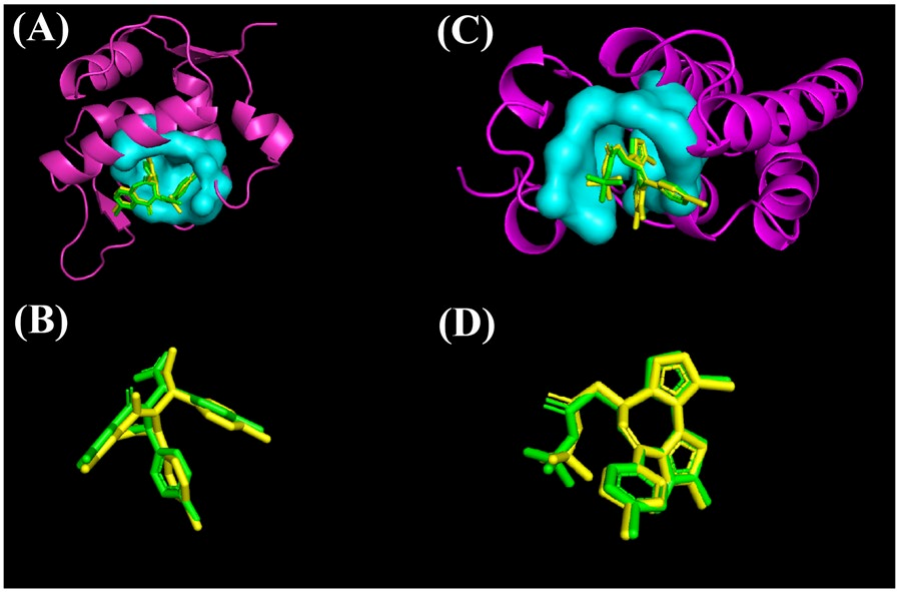
The binding site identification and pose prediction performance of CoBDock for two proteins, 1T4E and 3MXF. **A** and **C** represent the cavity and the ligand pose on proteins, 1T4E and 3MXF. The proteins are coloured by magenta, and the structure in cyan represents the cavity found as the top prediction. **B** and **D** represent the natural ligand and prediction of ligand pose, in green and yellow respectively

CoBDock achieved higher accuracy on the five datasets: ADS, MTi, DUD-E, CASF-2016 and PDBBind. Figure 10 also demonstrates an example of how successfully CoBDock not only finds cavities but also poses predictions by demonstrating pose prediction for 1T4E and 3MXF.

To determine the precise location and poses, CoBDock conducted a comprehensive search of the whole surface of 1T4E and 3MXF utilizing molecular docking programs and cavity detection techniques. Subsequently, the obtained 3D structural outcomes are transformed into vector representations using voxelization. The CoBDock model was utilized to rank the voxels of 1T4E and 3MXF in order to identify the most favourable cavity. The cyan pockets in Fig. 10 represent the locations of these promising cavities. Finally, the algorithm conducts a process of local docking using PLANTS in order to identify the poses depicted in Fig. 10.

CoBDock enables users to simultaneously manipulate the quantities of ligands and proteins, hence enhancing the practicality of the docking process. The software has the capability to perform docking of several ligands into various targets.

## Conclusion

The study presents Consensus Blind Dock (CoBDock), a pipeline designed to increase accuracy in blind docking by integrating molecular docking and cavity detection tools in parallel. The novel blind docking method, CoBDock, achieved 0.50$-$0.88% accuracy on different benchmark binding site studies. CoBDock outperformed P2Rank, Fpocket, CB-Dock and CB-Dock2 based on performance not only identification of binding site but also pose prediction. Also, the binding pose prediction accuracy (< 2 Å RMSD) of CoBDock is between 0.40$-$0.67%, the best results on five benchmarks.

As an end-to-end automated pipeline, CoBDock saves time and provides practical docking when a set of ligands are screening against a set of targets. The features of CoBDock expand blind docking applications, including target fishing, drug repositioning, and polypharmacological drug design. The performance of CoBDock will be investigated on these topics in further investigations.

CoBDock encompasses a total of four distinct molecular docking algorithms and two specialized tools for cavity detection. The performance of the system may be enhanced more effectively by increasing the number of components, as ablation Analysis results indicate that a pipeline with more components provides higher performance.

### Supplementary Information

**Additional file 1.** **Figure S11.** TM-score distribution between for benchmarks against training set. **Figure S12.** Selection of molecular docking program using CB-Dock and CoBDock predictedcoordinates. **Table S3** The summary of cavity detection tools used in the literature.

## Data Availability

Project Name: CoB-Dock. Project home page: https://github.com/DavidMcDonald1993/cobdock Dataset: PDBbind: http://www.pdbbind.org.cn/download.php DUD-E: https://seq2fun.dcmb.med.umich.edu//EDock/ Astex Diverse Set (ADS), and MTiOpenScreen Set (MTi): https://github.com/DavidMcDonald1993/cobdock. Operating system: Source code. Programming language: Python and Java. Other requirements: Autodock Vina, GalaxyDock3, PLANTS, ZDOCK, P2Rank and Fpocket. License: GNU GPL.

## Abbreviations

CoBDock: Consensus blind dock
ML: Machine learning
LBSs: Ligand binding sites
RMSD: Root-mean-square deviation
NMR: Nuclear magnetic resonance
EM: Electron microscopy
ADS: Astex Diverse Set
MTi: MTiOpenScreen Set
DUD-E: Directory of Useful Decoys Enhanced
PDB: Protein Data Bank
RMSD: The root-mean-square deviation of atomic positions
SVM: Support vector machines
KNN: K-nearest Neighbors Classifier
TPOT: A Tree-Based Pipeline Optimization Tool for Automating Machine Learning
CB-0: CB-Dock
CB-1: Structure-based blind docking of CB-Dock2
CB-2: Template-based blind docking of CB-Dock2
CB-C: Combining of all poses of CB-Dock2
