# Supplementary material for: Cobdock: an accurate and practical machine learning-based consensus blind docking method
Source: J Cheminform. 2024 Jan 11;16:5. doi: 10.1186/s13321-023-00793-x (PMC10785400; doi:10.1186/s13321-023-00793-x)
Supplement: Supplementary file 1 — Additional file 1. Figure S11. TM-score distribution between for benchmarks against training set. Figure S12. Selection of molecular docking program using CB-Dock and CoBDock predictedcoordinates. Table S3 The summary of cavity detection tools used in the literature. [file 13321_2023_793_MOESM1_ESM.pdf]

Supplementary information

The TM-Scores of the pairings derived from the training set and benchmarks

The TM-Scores were computed for pairings consisting of sequences from the training set and those from the benchmark datasets. Proteins in the training set are excluded after their protein pair achieves a TM-score greater than 0.5, in order to maintain the integrity of the benchmarks. Figure 11 illustrates the distribution pertaining to each benchmark, revealing a lack of resemblance between the training set and the benchmarks.

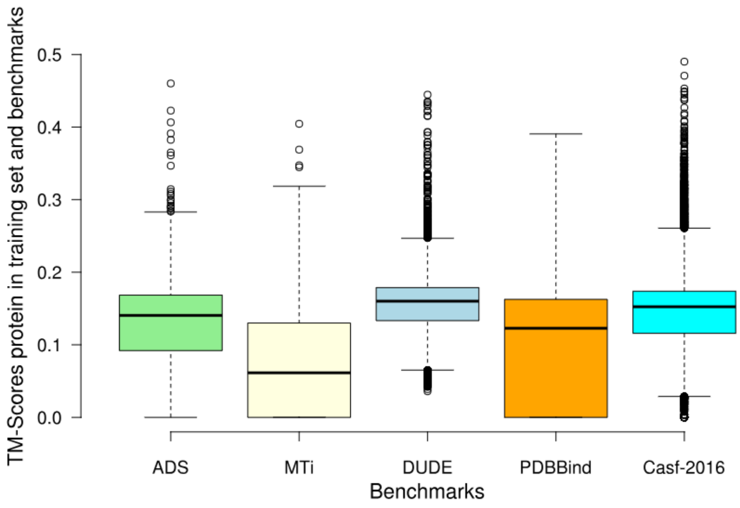

Figure 11 TM-score distribution between for benchmarks against training set

The TM-Score has been computed for each protein benchmark in order to exclude proteins that are redundant. Consequently, our model exhibited an inability to retain the structural information necessary for accurate predictions. Instead of relying on rote memorization of specific data paths, CoBDock uses a learning approach to derive insights and enhance its predictive capabilities.

Comparison of performance of CB-Dock and CoBDock across several molecular docking protocols

The enhancement in CoBDock performance can be attributed to the exceptional performance exhibited by the binding site. However, CB-Dock uses the Vina algorithm instead of using PLANTS, which may account for the superior performance of CoBDock over CB-Dock. Consequently, the centroids of the predicted binding sites of both CB-Dock and CoBDock are employed to execute three distinct molecular docking algorithms, namely GalaxyDock3, PLANTS, and Vina, on the ADS benchmark dataset.

The performance of CoBDock surpasses that of CB-Dock, even when employing distinct molecular docking algorithms, resulting in a substantial improvement.

Figure 12 illustrates that PLANTS exhibits superior pose-prediction performance when each molecular docking program is run with their respective default parameters and a search area of 15Ax15Ax15A. This performance provides evidence in favour of our decision to use PLANTS in the final stage of the CoBDock pipeline.

The table presented in this study (Table 3) provides an overview of the various cavity detection tools documented in the literature, excluding P2rank and Fpocket. DiffDock is a pipeline that is often discussed in the academic literature due to its competitive performance. However, it should be noted that DiffDock employs a deep learning technique, which may compromise the interpretability of the model. Therefore, Fpocket, CB-Dock, and CB-Dock2, P2rank is employed for comparison purposes.

CoBDock has great potential to be used as a "meta learner", which can learn from base programs, such as P2rank, and Fpocket. Hence, when more pipelines achieve success and are made publicly available, they can readily be integrated into CoBDock to further enhance performance. The ensemble model, in general, exhibits superior performance compared to individual base models such as DiffDock [65, 66].

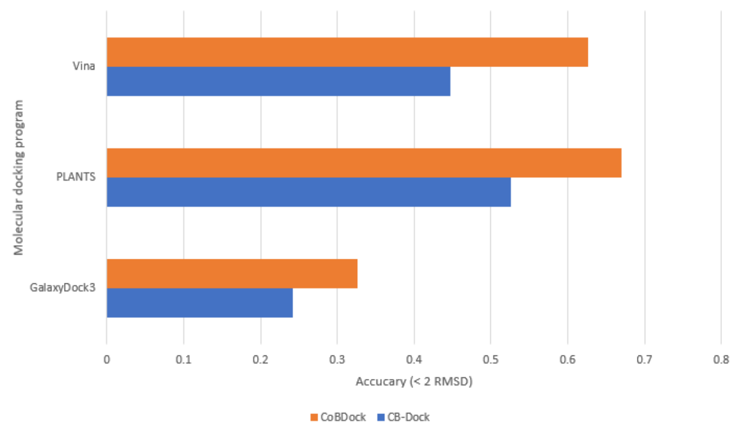

Figure 12 Selection of molecular docking program using CB-Dock and CoBDock predicted coordinates
The potential for CB-Dock and other pipelines to yield improved root-mean-square deviation (RMSD) values is limited, as their performance is already compromised at the binding site identification stage. However, the use of predicted coordinates from CB-Dock and CoBDock has been employed to carry out local docking in order to examine the influence of local docking programs on the performance of RMSD. In order to perform local docking, three distinct small molecule-protein docking tools were employed, namely GalaxyDock3, PLANTS, and Vina.

Table 3 The summary of cavity detection tools used in the literature

Cavity detection tool	Type	Overview
DiffDock	Deep learning	DIFFDOCK is a diffusion-generative model over the non-Euclidean manifold of ligand positions [67].
SiteHound	Energetic	Molecular Interaction Fields (MIFs) generated by EasyMIFs are used by SiteHound to pinpoint areas of protein structure that have a high propensity for ligand interaction [68].%
DeepSite	Machine learning	A completely machine-learning method for predicting protein-ligand-binding sites is called DeepSite [69].
Metapocket 2.0	Consensus	Metapocket2 uses Fpocket, GHECOM, ConCavity, and POCASA to improve their performance [21].

The identification of binding sites has been a challenge in the field of structural research, prompting the development of several binding site methods throughout the years. A subset of individuals were provided with a brief introductory overview.
